# Latent Class Analysis of eHealth Literacy in Patients with Acute Coronary Syndrome: Associations with Secondary Prevention Medication Adherence and Care Dependency

**DOI:** 10.12669/pjms.42.8.14802

**Published:** 2026-08

**Authors:** Mengxi He, Ru Ye, Linglong Chen, Aidi Pan

**Affiliations:** 1 Mengxi He, Department of Emergency, Wenzhou People’s Hospital, Wenzhou 325000, Zhejiang, China; 2 Ru Ye, Department of Emergency, Wenzhou People’s Hospital, Wenzhou 325000, Zhejiang, China; 3 Linglong Chen, Department of Emergency, Wenzhou People’s Hospital, Wenzhou 325000, Zhejiang, China; 4 Aidi Pan, Department of Emergency, Wenzhou People’s Hospital, Wenzhou 325000, Zhejiang, China

**Keywords:** Acute coronary syndrome, Care dependency, eHealth literacy, Latent classes, Secondary prevention medication adherence

## Abstract

**Objective:**

To investigate the latent classes of eHealth literacy in patients with acute coronary syndrome(ACS) and their associations with secondary prevention medication adherence and care dependency.

**Methodology:**

This was a retrospective study. A cross-sectional survey was conducted on 142 ACS patients admitted to the Emergency Department of Wenzhou People’s Hospital from January 2024 to November 2025, via convenience sampling method. Data were retrospectively extracted from the hospital’s electronic medical record(EMR) system. Latent class analysis (LCA), univariate analysis, and multivariate logistic regression analysis were applied to identify the latent classes of eHealth literacy and examine their associations with secondary prevention medication adherence and care dependency.

**Results:**

Three eHealth literacy subgroups were identified: low (23.24%), moderate (50.00%), and high (26.76%). Age, education, stress, insurance, medication adherence, and care dependency were significant influencing factors(P< 0.05).

**Conclusion:**

Acute coronary syndrome(ACS) patients showed heterogeneity in eHealth literacy latent classes. eHealth literacy was positively correlated with medication adherence and negatively correlated with care dependency, which were influenced by age, education, stress, insurance type, medication adherence and care dependency.

## INTRODUCTION

Acute coronary syndrome (ACS) is a life-threatening cardiovascular disease characterized by acute coronary artery occlusion leading to myocardial ischemia and necrosis. With acute onset, rapid progression, and high mortality, ACS remains a leading cause of global death and disability.^1^ Despite major advances in acute-phase management, long-term post-discharge care continues to pose a major clinical challenge. Approximately 30%–50% of ACS patients show poor adherence to secondary prevention, which increases the risk of recurrent cardiovascular events 2–3-fold and sustains high rates of readmission and mortality.^2^,^3^ Moreover, ACS patients often develop a “high dependency–low self-care” pattern due to physical limitations, psychological burden, and increased care needs,^4^ which further hinders rehabilitation and quality of life.

In this context, eHealth literacy has become a key factor in improving the prognosis of ACS patients. It refers to the ability to search, obtain, evaluate, and apply health information from electronic resources to solve health problems.^5^ Studies have shown that patients with higher eHealth literacy more actively use online medical resources,^6^ demonstrate better medication adherence, and have stronger self-management and decision-making capacity, thereby reducing care dependence. However, eHealth literacy among Chinese ACS patients is generally low,^7^ with many patients unable to effectively use digital tools due to inadequate digital skills, poor information evaluation ability, or low motivation.

ACS patients may have distinct eHealth literacy subgroups, but related evidence is limited. This study used latent class analysis to explore subgroups and their relationships with medication adherence and care dependency, providing evidence for targeted interventions.

## METHODOLOGY

This retrospective study included 142 patients with acute coronary syndrome (ACS) treated at the Emergency Department of Wenzhou People’s Hospital between January 2024 to November 2025. Data were retrospectively extracted from the hospital’s electronic medical record (EMR) system. The sample size met the minimum requirement of at least 137 cases, calculated based on 25 variables and allowing for a 10% attrition rate.

### Ethical approval:

The study was approved by the Institutional Ethics Committee of Wenzhou People’s Hospital (No.:KY-202510-041; Date: October 13, 2025), and written informed consent was obtained from all participants.

### Inclusion criteria:

- Definite diagnosis of acute coronary syndrome(ACS) according to relevant diagnostic criteria, confirmed by ECG changes (unstable angina, STEMI, NSTEMI), dynamic elevation of myocardial injury biomarkers (troponin I/T or CK-MB), and typical clinical manifestations (e.g., chest pain).
- Age ≥ 18 years.
- Clear consciousness (Glasgow Coma Scale [GCS] ≥ 15), able to communicate and complete the survey either independently or via researcher-assisted verbal inquiry.
- Disease stage: 1–6 months after discharge following ACS treatment (a critical phase for secondary prevention, with regular medication initiated and a stronger correlation between eHealth literacy and medication adherence).
- Informed consent: Voluntary participation in the study and provision of written informed consent.

### Exclusion criteria:

- Complicated with severe cognitive impairment (MMSE < 24) caused by dementia, severe stroke, etc., and inability to understand medication adherence or eHealth literacy items.
- Severe mental illness (e.g., schizophrenia, bipolar disorder) that may affect questionnaire validity or medication autonomy.
- Complete inability to use electronic devices, precluding completion of the eHealth literacy assessment;
- Early death after ACS admission, transfer to a facility unavailable for follow-up, or discontinuation of secondary prevention for other reasons.
- Receiving hospice/palliative care, or having discontinued all secondary prevention medications due to allergies or severe adverse reactions;
- Duplicate participation in similar studies on eHealth literacy or medication adherence.

### General information questionnaire:

The general information questionnaire included items on gender, age, educational level, marital status, self-reported stress in the past year, current living status, type of medical insurance, disease duration, and types of comorbidities.

### Coronary heart disease (CHD) patients’ eHealth literacy scale:

This scale was developed by Zhang S et al.^8^ in 2025, with 22 items covering four dimensions: functional (6), critical (6), interactive (4), and transformative eHealth literacy (6). It demonstrated excellent content validity (scale-level CVI = 0.875; item-level CVI = 0.882–1.000) and satisfactory internal consistency (Cronbach’s α = 0.915, 0.890, 0.819, 0.896 for each dimension, all >0.7). Scored on a 4-point Likert scale, higher scores indicated higher eHealth literacy. The Cronbach’s α of the total scale in this study was 0.75.

### CHD patients’ secondary prevention medication adherence scale:

This scale was developed by Zhang W et al.^9^, comprising six items. It demonstrated perfect content validity (scale- and item-level CVI = 1.00), good internal consistency (Cronbach’s α = 0.83) and test-retest reliability (0.87). Scored on a 4-point Likert scale (1=completely non-adherent, 2=non-adherent unless modified, 3=highly adherent but requiring modification, 4=completely adherent), higher scores indicated better secondary prevention medication adherence. The Cronbach’s α in this study was 0.81.

### Care dependency:

This scale was translated and culturally adapted into Chinese by Zhang S et al.^10^, comprising 15 items with an original Cronbach’s α of 0.95. It uses a 5-point Likert scale (1=completely dependent, 2=largely dependent, 3=partially dependent/independent, 4=mostly independent, 5=completely independent). Total scores range from 15 to 75, with lower scores indicating higher care dependency. The Cronbach’s α in this study was 0.87.

### Data collection methods:

After clinical stabilization, face-to-face interviews were conducted by trained investigators. Questionnaires were checked and completed on site. A total of 150 questionnaires were distributed; 8 were excluded, and 142 valid ones were included, with an effective recovery rate of 94.67%.

### Statistical analysis:

Data were entered into Microsoft Excel 2022. Latent class analysis was performed using Mplus 8.3 based on four eHealth literacy dimensions, with optimal model determined by fit indices including AIC, BIC, entropy, LMRT and BLRT. Statistical analyses were conducted using SPSS 26.0. The confidence interval is 95%. Data were presented as mean ± standard deviation or frequencies. One-way ANOVA, logistic regression and bivariate correlation were applied for related statistical analysis.

## RESULTS

Among 150 enrolled ACS patients, eight were lost to follow-up, yielding 142 valid cases. The sample included 53 females and 89 males with diverse sociodemographic and clinical characteristics.

Mean eHealth literacy score was 38.85±12.503. Bivariate correlation analysis showed eHealth literacy was positively correlated with medication adherence (r=0.251, P<0.001) and negatively correlated with care dependency (r=-0.451, P<0.001) (Table-I).

**Table-I:** eHealth Literacy Scores and Their Associations with Secondary Prevention Medication Adherence and Care Dependency in ACS Patients.

	Score Range	Mean Value	Standard Deviation	1	2	3	4	5	6	7
1 eHealth Literacy	22-82	38.85	12.503	1						
2 Functional eHealth Literacy	6-24	11.39	4.904	0.943**	1					
5 Transformative eHealth Literacy	6-22	12.11	9.418	0.930**	0.830**	0.812**	0.801**	1		
6 Secondary Prevention Medication Adherence	6-24	17.29	6.707	0.251**	0.232**	0.239**	0.273**	0.184**	1	
7 Care Dependency	15-70	40.33	19.187	-0.451**	-0.522**	-0.539**	-0.273**	-0.484**	-0.351**	1

**Fig.1:**
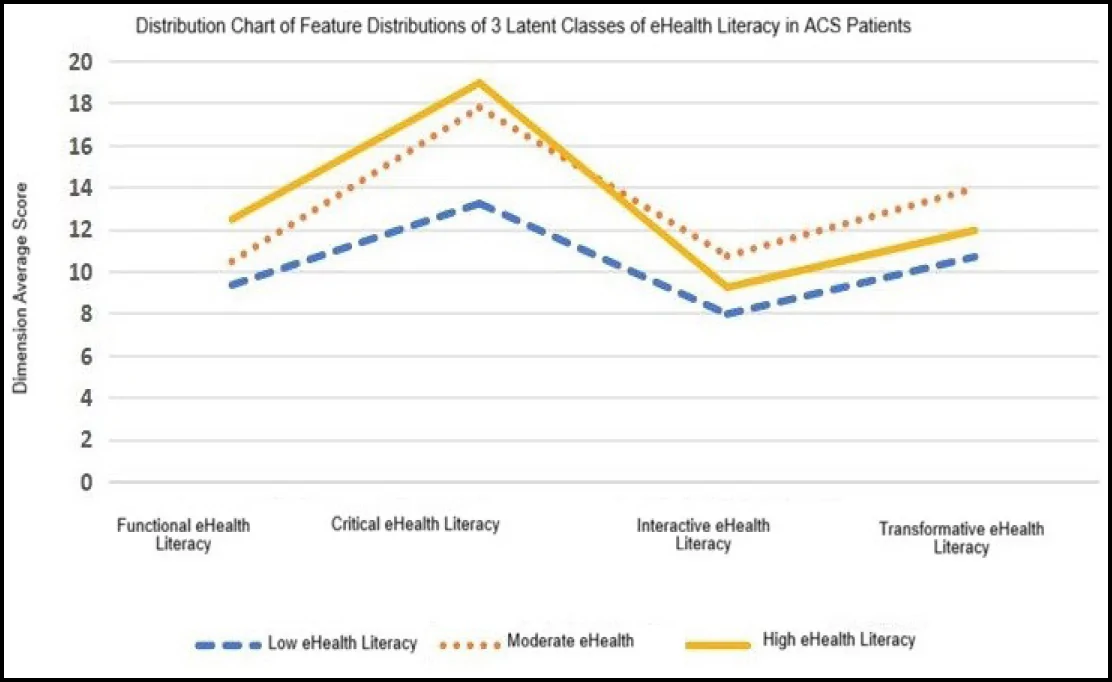
Characteristic Distribution of Three Latent Classes of eHealth Literacy in ACS Patients.

Latent class analysis supported a 3-class model: low (23.24%), moderate (50.00%), and high eHealth literacy (26.76%); model fit indices are shown in Table-II, and class distribution in Fig.1. Univariate analysis identified significant between-class differences in age, education, stress, insurance, comorbidities, medication adherence and care dependency (all P<0.05) (Table-III). Multivariate logistic regression confirmed these as independent influencing factors (Table-IV).

**Table-II:** Fit Analysis of Latent Class Models for eHealth Literacy in ACS Patients.

Model	AIC	BIC	aBIC	Entropy	P		Class Probability
					LMRT	BLRT	
1	112481.402	1226506.803	143373.361				1.000
2	100169.611	101126.615	111343.264	0.963	<0.001	<0.001	42.37/ 57.63
3	9572.945	92023.531	93340.286	0.984	<0.001	<0.001	23.24 /50.00/ 26.76
4	9322.247	90970.423	92167.276	0.872	0.056	0.113	15.67/ 25.34/ 35.89/ 23.08

***Note:*** AIC = Akaike Information Criterion; BIC = Bayesian Information Criterion; aBIC = Adjusted Bayesian Information Criterion; BLRT = Bootstrap Likelihood Ratio Test; LMRT = Lo-Mendell-Rubin Likelihood Ratio Test.

**Table-III:** Univariate Analysis of Factors Influencing Different Latent Classes of eHealth Literacy.

Item	Category	Low eHealth Literacy Type (n=33)	Moderate eHealth Literacy Type (n=71)	High eHealth Literacy Type (n=38)	(χ²/F)	P
Gender	Female	2 (6.06%)	27 (38.03%)	24 (63.16%)	8.055	1.013
	Male	31 (93.94%)	44 (61.97%)	14 (36.84%)		
Age	<40 years	8 (24.24%)	20 (28.17%)	15 (39.47%)	10.865	0.028
	40-60 years	20 (60.61%)	40 (56.34%)	16 (42.11%)		
	≥60 years	5 (15.15%)	11 (15.49%)	7 (18.42%)		
	Other	5 (15.15%)	16 (22.54%)	8 (21.05%)		
Disease Duration	<1 year	3 (9.09%)	14 (19.72%)	7 (18.42%)	13.971	0.044
	1-5 years	6 (18.18%)	14 (19.72%)	13 (34.21%)		
	6-10 years	14 (42.42%)	32 (45.07%)	5 (13.16%)		
	More than 10 years	10 (30.30%)	11 (15.49%)	13 (34.21%)		
Number of Comorbidities	0 comorbidities	4 (12.12%)	13 (18.31%)	3 (7.89%)	17.227	0.006
	1 comorbidity	6 (18.18%)	24 (33.80%)	10 (26.32%)		
	2 comorbidities	7 (21.21%)	16 (22.54%)	14 (36.84%)		
	≥ 3 comorbidities	15 (45.45%)	14 (19.72%)	11 (28.95%)		
Care Dependency		40.14±2.34	47.67±3.45	51.90±4.56	F=16.343	0.031

**Table-IV:** Logistic Regression Analysis of Latent Classes of eHealth Literacy in ACS Patients.

Comparison Group	Variable	Regression Coefficient	Standard Error	P value	OR value	95%CI	
C2 VS C1	Constant	-14.583	1.914	0.000			
	Secondary Prevention Medication Adherence Score	0.015	0.104	0.000	1.015	1.007	1.022
	Care Dependency Score	0.234	0.042	0.000	1.264	1.210	1.320
C3 VS C1	Constant	-25.184	6211.586	0.037			
	Secondary Prevention Medication Adherence Score	0.029	0.005	0.000	1.030	1.020	1.040
	Medical Insurance Type (Employee Medical Insurance)	0.622	0.721	0.046	1.075	0.379	0.662

***Note:*** C1 = Low eHealth Literacy Type; C2 = Moderate eHealth Literacy Type; C3 = High eHealth Literacy Type.

## DISCUSSION

In this study, ACS patients had a mean eHealth literacy score of 38.85±12.503, higher than Yan et al.’s report but still moderate.^11^ The four-dimension scores showed deficiencies in accessing, appraising, and applying digital health information, mainly due to poor digital tool use and limited self-management translation. Targeted interventions such as personalized guidance and health education are needed.

Latent class analysis identified three distinct subgroups of eHealth literacy among ACS patients: low (C1, n=33, 23.24%), moderate (C2, n=71, 50.00%), and high (C3, n=38, 26.76%), confirming significant population heterogeneity.^12^ Patients in the low-literacy class exhibited uniformly low item-response probabilities across all dimensions, suggesting substantial barriers in acquiring and applying electronic health information. Those in the moderate class demonstrated basic competencies but lacked proficiency in specific areas. Individuals in the high-literacy class achieved the highest response probabilities, yet still showed room for improvement in certain subdomains. These findings underscore the necessity of developing literacy-level-specific interventions—basic digital skill training for the low group, critical appraisal and interactive skill enhancement for the moderate group, and advanced health information integration for the high group—to optimize disease self-management and clinical outcomes.

Univariate and multivariate logistic regression analyses revealed that age, educational level, self-reported stress over the past year, medical insurance type, secondary prevention medication adherence, and care dependency were independent factors associated with latent class membership. These results are consistent with prior studies.^13^-^18^ Age remains a critical determinant, as older patients often face greater challenges in adopting digital technologies and processing health information.^19^ Educational attainment is positively associated with eHealth literacy, reflecting differences in information comprehension and problem-solving skills. Self-reported stress may impair patients’ motivation and cognitive capacity to engage with digital health resources.^19^ Insurance type, as a proxy for socioeconomic status, influences both access to care and health information-seeking behavior.^20^ Furthermore, a bidirectional relationship may exist between eHealth literacy and both medication adherence and care dependency: patients with higher eHealth literacy are more likely to comprehend medical instructions and adhere to treatment, thereby reducing dependency on caregivers; conversely, better adherence and lower care dependency may create a more favorable environment for acquiring eHealth skills. These findings highlight the importance of integrating psychosocial and structural factors into the design of personalized eHealth literacy interventions.

### Limitations:

First, the single-center design, with participants recruited exclusively from a tertiary hospital in Zhejiang Province, may limit the generalizability of the findings. Second, the cross-sectional nature of the study precludes causal inferences. Third, eHealth literacy was assessed via self-report, which may be subject to social desirability and recall biases.

## CONCLUSIONS

Latent class analysis identified three distinct eHealth literacy classes among ACS patients: low, moderate, and high. eHealth literacy was positively associated with medication adherence and negatively with care dependency. Influencing factors included age, education, stress, insurance type, medication adherence, and care dependency.

### Recommendations:

Future multicenter longitudinal studies with objective literacy measures are warranted to validate and extend our findings.

### Authors’ Contributions:

**MH** and **RY:** Conceived, designed, Manuscript writing and are responsible for the integrity of the study.

**LC** and **AP:** manuscript drafting, clinical data collection and analysis.

All authors have read and approved the final manuscript.
